# Supplementary material for: Sex Differences in Neuropathy: The Paradigmatic Case of MetFormin
Source: Int J Mol Sci. 2022 Nov 22;23(23):14503. doi: 10.3390/ijms232314503 (PMC9738696; doi:10.3390/ijms232314503)
Supplement: Supplementary file 1 [file ijms-23-14503-s001.zip › ijms-2028952-supplementary.pdf]

Article

Sex differences in Neuropathy: the paradigmatic case of metformin

Federica De Angelis [1,2], Valentina Vacca [1,2], Jessica Tofanicchio [3,+], Georgios Strimpakos [1], Giacomo Giacovazzo [2], Flaminia Pavone [1], Roberto Coccurello [2,4,*] and Sara Marinelli [1,*]

[1] National Research Council (CNR) Institute of Biochemistry and Cell Biology, Monterotondo (RM), Italy

[2] Sapienza University of Rome, Neurobiology student; Current address: SPA Società Prodotti Antibiotici S.p.A. Milan, Italy

[3] National Research Council (CNR) Institute for Complex System (ISC);

[4] European Center for Brain Research - IRCCS Santa Lucia Foundation, Rome Italy

* Correspondence: RC roberto.coccurello@cnr.it (R.C.); sara.marinelli@cnr.it (S.M.)

Supplemantary materials

Supplementary Figure S1

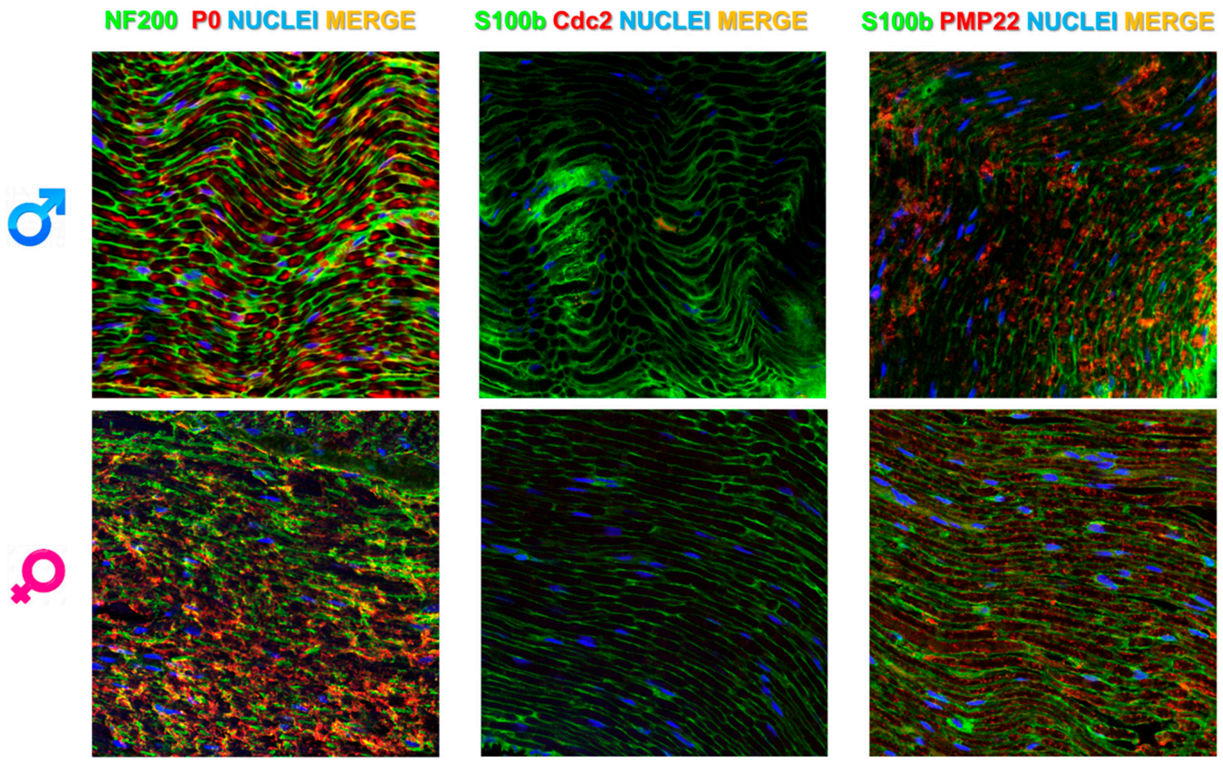

Supplementary Figure S1- Expression of neurofilament (NF200), myelin protein 0 (P0), S100beta, Cyclin-dependent kinase 1 (Cdc2), peripheral myelin protein 22 (PMP22) in sciatic nerve of male and female naïve mice.
